# Evaluation of Epigallocatechin-3-gallate Modified Collagen Membrane and Concerns on Schwann Cells

**DOI:** 10.1155/2017/9641801

**Published:** 2017-08-15

**Authors:** Chenyu Chu, Jia Deng, Cong Cao, Yi Man, Yili Qu

**Affiliations:** ^1^State Key Laboratory of Oral Diseases, West China Hospital of Stomatology, Sichuan University, Chengdu 610041, China; ^2^China-Japan Friendship Hospital, Department of Stomatology, Beijing 100029, China; ^3^Department of Oral Implantology, West China Hospital of Stomatology, Sichuan University, Chengdu 610041, China

## Abstract

Collagen is an essential component of the extracellular matrix (ECM) and is a suitable material for nerve repair during tissue remodeling for fracture repair. Epigallocatechin-3-gallate (EGCG), an extract of green tea, shows various biological activities that are beneficial to nerve repair. Here, we developed modified collagen containing different concentrations of EGCG (0.0064%, 0.064%, and 0.64%, resp.) to induce Schwann cell proliferation and differentiation. Cell Counting Kit-8 test, live/dead assay, and SEM showed that collagen cross-linked by EGCG induced Schwann cell proliferation. Real-time polymerase chain reaction, enzyme-linked immunosorbent assay, and Western blotting revealed that EGCG-modified collagen induced Schwann cell differentiation and downregulated reactive oxygen species (ROS) levels by downregulating the MAPK P38 signaling pathway. Our results indicate that collagen cross-linked with an appropriate concentration of EGCG induces the proliferation and differentiation of Schwann cells. The EGCG-modified collagen membrane may be applicable for nerve repair and guided tissue regeneration applications.

## 1. Introduction

Peripheral nerve regeneration is in a debilitating condition for which new bioengineering solutions are needed [1]. Autografting is the current gold standard treatment for nerve repair but is limited by the availability of expendable donor nerves, resulting in a second injury with the loss of sensation at the donor site [2, 3]. Moreover, the nervous system is involved in bone remodeling after bone fracture [4]. It regulates bone regeneration by releasing related peptides, such as calcitonin gene-related peptide, neuropeptide Y, and intestinal peptide [5]. Thus, nerve regeneration critically influences the success of guided tissue regeneration (GTR) treatment.

Recently, GTR biomaterials and cells have been developed for nerve repair. Extracellular matrix (ECM) molecules and Schwann cells (SCs) are important components of peripheral nerve repair. Armstrong et al. showed that ECM molecules affected SC behaviors, including attachment, proliferation, and secretion of neurite-promoting factors by SCs on a nerve conduit polymeric material* in vitro*.

Collagen is an essential component of the ECM, shows excellent biocompatibility, and is beneficial for a series of cellular behaviors including cell adhesion, proliferation, migration, and differentiation [6–8]. Collagen is widely used in nerve repair. Macaya et al. investigated the proproliferative effect of injectable collagen-based hydrogels by incorporating fibroblast growth factor-2 on astrocytes, which plays an important role in the healing of spinal cord injury [9]. Another study by Cui et al. involved the use of collagen scaffolds incorporated with the neurocytokines ciliary neurotrophic factor and basic fibroblast growth factor to bridge a nerve gap [10]. Moreover, Sadtler et al. applied an ECM-based collagen membrane to guide tissue regeneration by shaping the immune microenvironment. These studies indicated that an ECM-based collagen membrane mediates macrophages and T helper cells to promote systemic and local proregenerative immune responses [11]. Additionally, collagen was shown to promote nerve reconstruction. However, as a foreign material to the body, pure collagen may induce inflammatory responses, greatly limiting its biomedical applications.

Epigallocatechin-3-gallate (EGCG) is an important component of tea and has been reported to have advantageous biological activities including antioxidant [12] and anti-inflammatory effects [13, 14]. Previous studies have suggested that EGCG protects nerve cells from oxidative-radical-stress-induced apoptosis [15], has anti-inflammatory and neuroprotective potential [16], modulates neurological function [17], activates nerve growth factors to induce neuritogenesis and reduce neuropathic pain after chronic constriction nerve injury [18], and stimulates regeneration of myelinated axons and minimizes histomorphological alterations caused by crush injury of the sciatic nerve [19].

Thus, EGCG-treated collagen may be useful in nerve repair and GTR [20, 21]. In this study, we fabricated an EGCG-modified collagen scaffold to accelerate tissue repair, trigger SC proliferation and differentiation, and induce secretion of nutrient growth factors. Cell viability, the levels of nutrient factors, including nerve growth factor (NGF) and brain-derived neurotrophic factor (BDNF), and the expression of Krox-20 in RSC96 cells were also measured to assess the biological properties of EGCG-treated collagen membranes.

## 2. Methods and Materials

### 2.1. Materials

Each type I collagen membrane (Zhenghai Biotechnology, Shandong, China) was commercially available and was prepared in a size of 10 mm diameter. The collagen membrane was made of natural and untreated ECM that maintains its structure and characteristics well. EGCG was obtained commercially (Jiang Xi Lv Kang Natural Products, Jiang Xi, China). All solvents and chemicals were of analytical grade and used without further purification.

### 2.2. Fabrication of EGCG-Modified Collagen

EGCG was dissolved in deionized water to prepare EGCG solutions of 0.64%, 0.064%, and 0.0064% (w/v) according to the previous study [22]. Then, collagen membranes were immersed in different concentrations of EGCG at room temperature for 1 h to fabricate the EGCG-modified collagen membranes. After being immersed, the resulting collagen membranes were rinsed three times with deionized water and then freeze-dried overnight. A control group was incubated with deionized water under the same experimental conditions.

### 2.3. Cell Viability

Cell Counting Kit-8 (Dojindo Laboratories, Kumamoto, Japan) was applied to detect cell viability. The cells were cultured in DMEM with 10% FBS. All the pure and the EGCG-modified collagen membranes were placed in 48 well plates where RSC96 cells of 10^4^/well were seeded. After seeding, 10% of CCK-8 solution was added to each well, and then the 48 well plates were continuously incubated at 37°C for 2 h. After incubation, the absorbance of the solution was read at 450 nm.

### 2.4. Live/Dead Cell Assay

To further investigate the viability of cells, a live/dead cell assay kit (Dojindo Laboratories, Kumamoto, Japan) was applied according to the manufacturer's instructions. RSC96 cells were cultured on various collagen membranes for 5 days before being examined. Membranes with cells seeded were washed with 1x PBS for 5 min and incubated with Calcein-AM 2 *μ*M plus EthD-1 4 *μ*M for 15 min at 37°C in the dark. After incubation, the resulting samples were then washed with 1x PBS for 5 min and then analyzed by an Inverted Ti–E microscope (Nikon, Japan).

### 2.5. Scanning Electron Microscope (SEM) Observation

SEM (S-800, HITACHI, Tokyo, Japan) with an accelerating voltage of 25 kV was used to observe the morphologies of collagen membranes. In order to achieve enough electrical conductivity, all the samples were coated with an ultrathin layer (300 Å) of Au/Pt in an ion sputter (E1010, HITACHI, Tokyo, Japan).

### 2.6. Immunostaining of Cells

For immunofluorescence staining of F-actin (cytoskeleton), Krox-20 (differentiation marker of Schwann cells), and nucleus, the cells on the samples were fixed in 2% paraformaldehyde in PBS for 5 min (pH 7.4) followed by being washed three times in PBS (5 min). Then, the samples were pretreated with 1% bovine serum albumin in PBS containing 0.1% Triton X-100 (1 h) and then incubated in 1% Tween 20 for 20 min. After incubation, the samples were given a 5 min wash in PBS, and then cells were covered by sufficient stain solution and incubated for 1–5 min in the dark. All of antibodies were purchased from Santa Cruz Biotechnology (Texas, USA).

After removing the stain solution by washing the cells 2-3 times in PBS, fluorescent images were collected.

### 2.7. Neurotrophins Determination by Real-Time Polymerase Chain Reaction (RT-PCR) and Enzyme-Linked Immunosorbent Assay (ELISA)

RSC96 cells were seeded on the pure collagen and collagen loaded with EGCG of different concentrations and then incubated for 7 and 14 days. Total RNA was extracted with TRIZOL reagent and reverse transcribed using the mRNA Selective PCR kit. Rat NGF and BDNF cDNA were amplified by real-time PCR using the SYBR Green PCR kit. The primer sequences used for the real-time PCR are shown in Table 1. Moreover, levels of growth factors in the supernatants were determined using an ELISA kit. According to the manufacturer's instructions, the levels of these neurotrophins were analyzed using an Opt ELISA kit (BD Biosciences, San Jose, CA, USA). The absorbance (450 nm) was measured in a microplate reader.

### 2.8. Western Blot Analysis

To obtain whole cell protein extracts, RSC96 cells at day 7 were collected in cell lysis buffer. Whole cell extracts were sonicated and centrifuged to obtain the total proteins. Total protein in each sample was quantitated and concentrations were obtained using the BCA assay (Keygene, Nanjing, China). For Western blot analysis, 25 *μ*g of proteins was electrophoretically separated by 10% SDS-polyacrylamide gel electrophoresis and transferred to nitrocellulose membranes. The membranes were blocked with 5% bovine serum albumin and incubated with 1–5 *μ*g of primary antibodies against Krox-20 and p38/phosphor-p38 MAPK. Bound proteins were visualized with HRP-conjugated anti-rabbit IgG for Krox-20, p38, and phospho-p38. Enhanced chemiluminescence assay was used for the visualization of the protein bands. Moreover, ImageJ was used for the gray level difference analysis of results of Western blot (*N* = 3).

### 2.9. Statistical Analysis

All results are presented as mean value ± standard deviation. Differences between groups were analyzed by analysis of variance (one-way ANOVA) followed by Tukey's multiple comparison test (*α* = 0.05) by statistic software GraphPad Prism 5.

## 3. Results

### 3.1. Cell Viability and Cell Adhesion

**Figure 1:**
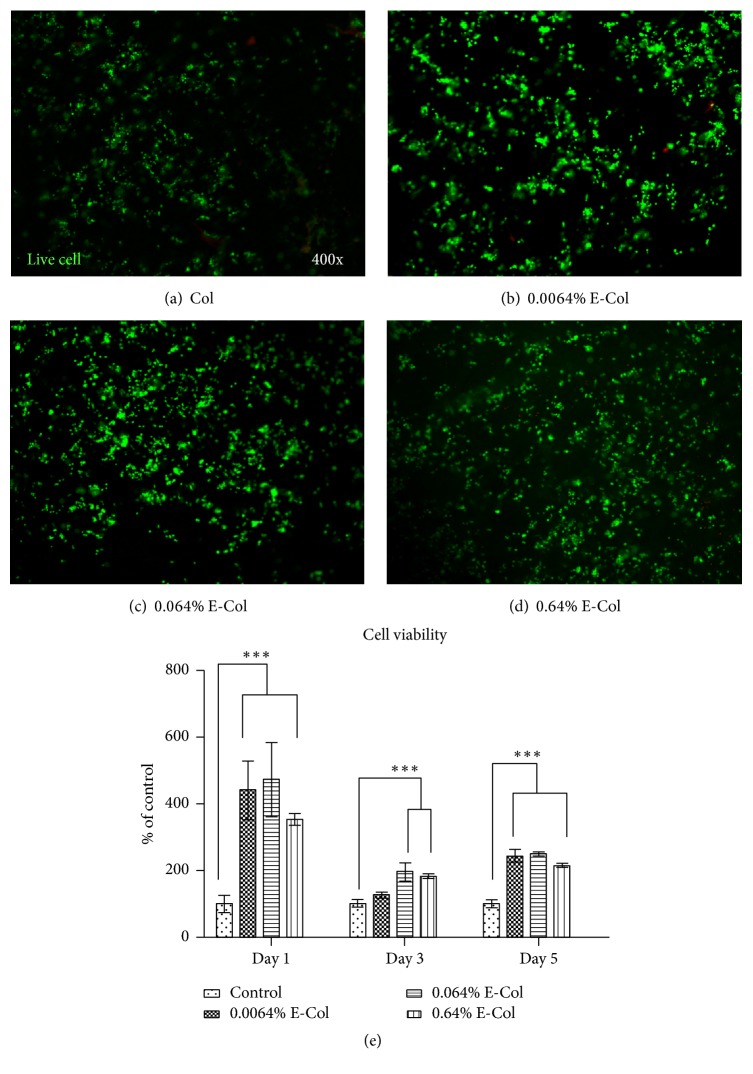
Live staining of RSC96 cells cultured on collagen membranes treated with 0% EGCG (a), 0.0064% EGCG (b), 0.064% EGCG (c), and 0.64% EGCG (d) on day 5. Live cells appear as green. (e) CCK-8 results of RSC96 cells cultured on various collagen membranes for 1, 3, and 5 days. Culture of different cells of collagen membrane treated or not treated with EGCG revealed the viability of different types of cells. ^*∗∗∗*^Significant difference compared to control group (Col) at *P* < 0.001.

**Figure 2:**
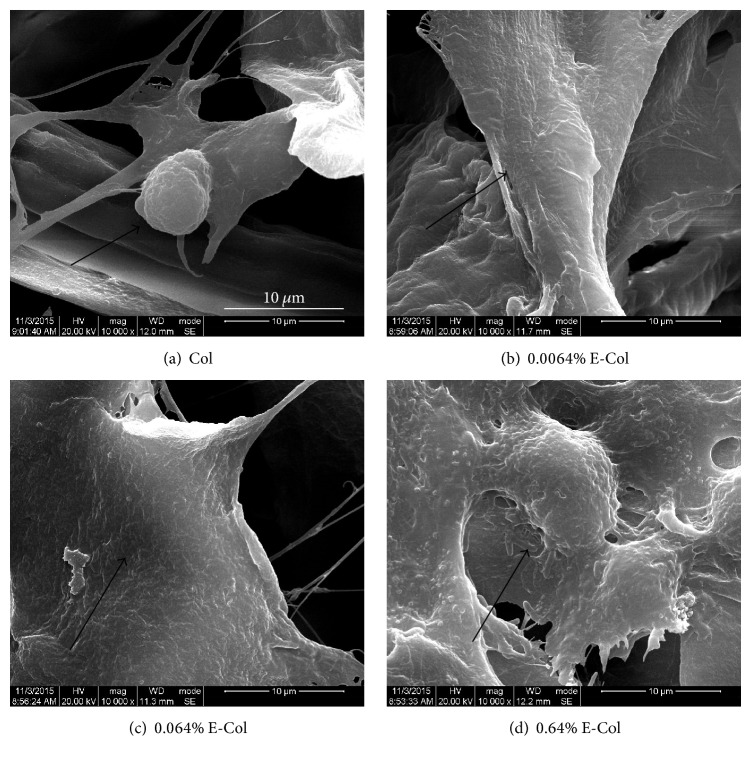
SEM images of the morphologies of RSC96 cells adhered to collagen membrane surfaces treated with 0% EGCG (a), 0.0064% EGCG (b), 0.064% EGCG (c), and 0.64% EGCG (d) after 24 h. 0.064% EGCG-collagen showed better morphology of RSC96 cells among the experimental groups, indicating higher cell proliferation. The black arrows indicate the cell-surface adhesion.

Cell viability was assessed with CCK-8 and live cells were stained with Calcein-AM.  Figure 1(e) shows the results of CCK-8 for RSC96 cells seeded on collagen on days 1, 3, and 5. Cell viability improved significantly after EGCG addition, whereas 0.064% EGCG-collagen showed the best effect on promoting proliferation in the experimental groups. In addition, cell viability after addition of 0.064% EGCG-collagen increased by nearly 5-fold compared to that of the control group on day 1. The images in Figures 1(a)–1(d) were obtained after seeding of RSC96 cells on collagen, with staining carried out on day 5. Live cells were stained green. More live cells were observed on collagen following treatment with the lower concentration of EGCG, whereas the highest concentration of EGCG showed more live cells compared to the control group. SEM analysis (Figure 2) revealed the morphologies of RSC96 cells adhered to different collagen membrane surfaces after 24 h. In EGCG-treated collagen membranes, the RSC96 cells were flatly spread across the sample surfaces. These results indicate that EGCG-collagen promotes the proliferation and cell viability of RSC96 cells.

### 3.2. Inducing Proliferation and Differentiation of SCs

**Figure 3:**
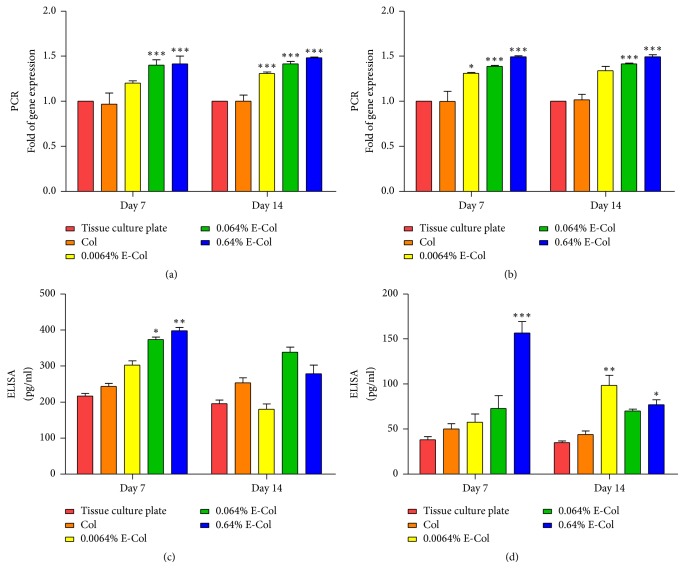
Gene expression of BDGF (a) and NGF (b) in RSC96 cells incubated on EGCG-treated collagen membranes for 7 and 14 days measured by RT-PCR. Quantification of released BDGF (c) and NGF (d) for 7 and 14 days produced by RSC96 cells measured by ELISA. ^*∗*,*∗∗*,*∗∗∗*^Significant difference compared to control group (Col) at *P* < 0.05, *P* < 0.01, and *P* < 0.001. According to ELISA and RT-PCR, neurotrophic factors produced by RSC96 cells were significantly increased after implantation of EGCG-modified collagen membranes.

The levels of neurotrophic factors were associated with improvements in survival, regeneration, differentiation, and synaptogenesis of neural fibers. Figures 3(a)–3(d) show that EGCG-treated collagen significantly increased the expression of neurotrophic factors (BDNF and NGF) secreted by RSC96 cells cultured on collagen, which increased with increasing concentration of EGCG according to RT-PCR and ELISA.

**Figure 4:**
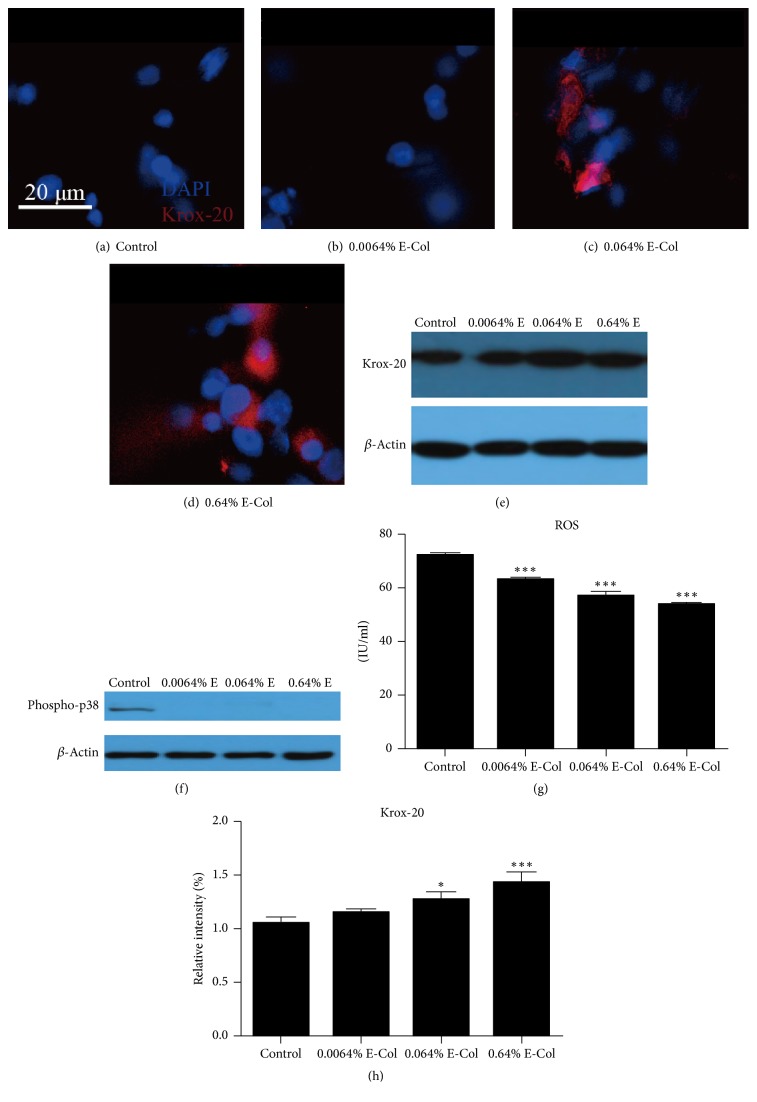
Immunofluorescence staining of Krox-20 (red) and DAPI (blue) in RSC96 cells on collagen membranes treated with 0% (a), 0.0064% (b), 0.064% (c), and 0.64% (d) EGCG. Compared to the collagen membranes in the control group (a), the collagen membranes treated with different concentrations of EGCG showed increased expression of Krox-20 (f). (e–g) Results of Western blot assay, indicating that EGCG-treated collagen induced Krox-20 expression and decreased ROS levels in RSC96 cells. (h) After gray level difference analysis of Krox-20, the gray levels of 0.64% E and 0.064% E were significantly higher than that of the control group. ^*∗*^*P* < 0.05; ^*∗∗∗*^*P* < 0.001, one-way ANOVA with Tukey's multiple comparison test.

SC differentiation into a myelinating phenotype requires several transcription factors [23], including Krox-20 proteins which directly regulate myelin protein expression [24, 25]. Figures 4(a)–4(d) show the immunostaining results of Krox-20 proteins, which stained as red, while the nucleus stained as blue, indicating that the expression of Krox-20 was increased after EGCG addition.  Figure 4(e) shows the results of Western blotting for Krox-20, which also supported increased expression, as the gray levels of Krox-20 in 0.64% E and 0.064% E were significantly higher than that of the control group. EGCG-modified collagen membranes downregulated P38 MAPK signaling pathways. Phospho-p38 was not activated in the experimental groups but activated in the control group according to Western blotting results (Figure 4(f)). Moreover, reactive oxygen species (ROS) levels were significantly decreased following addition of EGCG. In addition, ROS levels decreased with increasing concentrations of EGCG. Thus, EGCG-treated collagen induced SC differentiation and downregulated ROS levels by downregulating the MAPK P38 signaling pathway.

## 4. Discussion

Because of its biological compatibility and low immunogenicity, an increasing number of studies have shown that collagen can be used to fabricate biomaterials and is useful for nerve reconstruction [9, 10]. Previous studies have examined the application of EGCG in nerve repair [15–19]. In this study, EGCG-treated collagen induced the proliferation and differentiation of SCs. According to previous studies [22], collagen treated with EGCG showed better mechanical properties and the ability to regulate inflammatory factors, making it a suitable biomaterial for clinical applications.

The CCK-8 and live/dead assay revealed that RSC96 cells treated with EGCG-collagen showed higher cell viability than cells treated with pure collagen. Previous studies suggested that EGCG protects nerve cells from oxidative-radical-stress-induced apoptosis [15]. Interestingly, in the present study, the viability of RSC96 cells significantly increased with increasing concentrations of EGCG, including 0.64% EGCG, compared to the control group. However, in a previous study, the cell viability of osteoblasts was decreased when the cells were cultured on collagen membranes modified with 0.64% EGCG [26]. Based on these results, EGCG may have different effects on various cell types. Therefore, the appropriate concentration of EGCG for promoting cell viability varies in different cell types of cells; further studies are needed to confirm this result.

To successfully regenerate nerves, a disconnected nerve must bridge the gap between the proximal and distal stumps, following the SC tube to restore the function of sensory and/or motor targets [3]. Neurotrophic factors support this critical process and improve the survival, regeneration, differentiation, and synaptogenesis of neural fibers after injury. However, it remained unknown whether EGCG cross-linked collagen triggers SC proliferation and differentiation. Therefore, PCR and ELISA were performed to investigate the secretion of neurotrophic factors. The results showed that the expression of neurotrophic factors secreted by RSC96 cells cultured on EGCG-treated collagen were higher than that for untreated collagen, indicating that SCs proliferated and differentiated.

ROS play a deleterious role in the wound healing process, which can be associated with tissue injury. Excessive formation of free radicals can induce cell senescence and apoptosis [27]. The mitogen-activated protein kinase (MAPK) signaling pathway, which is involved in the production of proinflammatory growth factors, plays an important role in signal transduction from the surface to the nucleus of cell [28]. Excess inflammatory factors in the surgical site may lead to complications around implant biomaterials, causing regeneration failure [29, 30]. According to our experiment results, the EGCG-modified collagen membrane downregulated the levels of ROS. Moreover, the membrane inhibited activation of the MAPK P38 signaling pathway caused by ROS.

The differentiation of SCs into a myelinating phenotype requires several transcription factors, including Krox-20, a zinc-finger transcription factor essential for peripheral myelination [31]. Krox-20 also directly regulates myelin protein expression [24, 25]. In the present study, expression of Krox-20, determined by immunostaining, increased after the addition of EGCG in a dose-dependent manner, further indicating the differentiation of SCs into a myelinating phenotype. The increased expression of Krox-20 was also detected by Western blotting; the gray levels of 0.64% E and 0.064% E were significantly higher than that of the control group. These results indicate that EGCG-modified collagen membranes are innovative immune-mediated biomaterials that can be used to guide tissue regeneration.

## 5. Conclusions

In this study, collagen cross-linked by EGCG showed smooth surfaces and more uniform fibers. In addition, collagen membranes treated with appropriate concentration of EGCG can improve SC proliferation, differentiation, and migration and downregulate ROS levels by downregulating the MAPK p38 signaling pathway, indicating their potential for use in GTR treatment. This material may be used in nerve repair and numerous other clinical applications.

## Figures and Tables

**Table 1:** Nucleotide primers used for qRT-PCR.

Genes	Oligonucleotide sequence (5′-3′)
Gapdh	Forward: CCGTATCGGACGCCTGGTTA Reverse: CCGTGGGTAGAGTCATACTGGAAC
BDNF	Forward: TCTACGAGACCAAGTGTAATCCCA Reverse: CTTATGAACCGCCAGCCAAT
NGF	Forward: CTGGGCGAGGTGAACATTAACA Reverse: CAGCCTGTTTGTCGTCTGTTGTC

## Additional Points

*Highlights*. EGCG-treated collagen membrane significantly induces RSC96 cell proliferation and differentiation* in vitro*. EGCG-treated collagen membrane significantly induces secretion of neurotrophic factors in RSC96 cells. EGCG-treated collagen membrane downregulates ROS level in RSC96 cells by downregulating MAPK P38 signaling pathway.
